# Stemless shoulder prosthesis versus conventional anatomic shoulder prosthesis in patients with osteoarthritis: A comparison of the functional outcome after a minimum of two years follow-up

**DOI:** 10.1007/s10195-012-0216-9

**Published:** 2012-11-09

**Authors:** Alexander Berth, Géza Pap

**Affiliations:** 1 Department of Orthopaedics, Otto-von-Guericke-University, 39120 Magdeburg, Germany; 2 Centre of Orthopaedics and Traumatology, Park-Hospital Leipzig, 04289 Leipzig, Germany; 3 Orthopädische Universitätsklinik, Universitätsklinikum A. ö. R., Leipziger Str. 44, 39120 Magdeburg, Germany

**Keywords:** Stemless shoulder prosthesis, Shoulder, Arthroplasty, Osteoarthritis

## Abstract

**Background:**

The stemless shoulder prosthesis is a new concept in shoulder arthroplasty. To date, only a few studies have investigated the results of this prosthesis. The aim of this study was to investigate the clinical and radiological midterm results of this implant in comparison with a standard anatomic stemmed shoulder prosthesis.

**Materials and methods:**

The Constant score, the DASH score, the active range of motion (abduction, anteversion, external rotation), and the radiological results were examined in 82 patients with primary osteoarthritis of the shoulder treated with either the Total Evolutive Shoulder System^®^ (Biomed, France) stemless shoulder prosthesis or the Affinis^®^ (Mathys, Switzerland) stemmed shoulder prosthesis to detect possible differences in the functional outcome and to evaluate radiological properties of the implants. Patients were examined before and 32 ± 4 months after surgery.

**Results:**

There was no significant difference in the Constant scores of the groups treated with the stemless shoulder prosthesis (65.0 ± 11.0 points) and the stemmed shoulder prosthesis (73.2 ± 11.3 points; *P* = 0.162). The estimated blood loss (*P* = 0.026) and the mean operative time (*P* = 0.002) were significantly lower in the group with the stemless shoulder prosthesis.

**Conclusions:**

The use of the stemless shoulder prosthesis yielded good results which, in a mid-term follow-up, were comparable with those provided by a standard anatomic shoulder prosthesis. Further investigations are needed regarding the long-term performance of this prosthesis.

## Introduction

Shoulder arthroplasty is widely used in the treatment of severe osteoarthritis of the glenohumeral joint to relieve pain and to restore shoulder function [1, 2]. Many factors contribute to the outcome of shoulder arthroplasty, including the primary indication, the stiffness of the soft tissue, the status of the rotator cuff, previous surgeries, the preoperative range of motion, and the postoperative rehabilitation program [3]. Another influential factor for the postoperative functional results is the reconstruction of the physiological articulation and kinematics of the glenohumeral joint [4]. Therefore, modern concepts in shoulder arthroplasty have recently focused on the exact restoration of the center of rotation of the glenohumeral joint [5–11]. These modern generations of shoulder implants (third- and fourth-generation designs) allow multidimensional adjustable adaption of the prosthesis in order to achieve the exact restoration of the anatomy of the proximal humerus. The combination of these modern humerus implants with glenoid replacement implants of appropriate size that are correct positioned have raised the expectation of a good functional outcome as well as long survivorship of the shoulder arthroplasty [1, 12–15]. Despite these efforts, the use of conventional shoulder prostheses is still associated with potential problems related to the stem, such as periprosthetic fractures and malpositioning or loosening of the humeral component [16–18]. Therefore, the stemless shoulder prosthesis was introduced as a new modern shoulder replacement system that was designed to reduce the potential risks associated with using a stemmed humeral implant. Today, a few types of stemless shoulder implants are available, but only a few studies have reported the clinical results of using them [19–21]. Therefore, the purpose of the study described in this paper was to evaluate the clinical and radiological midterm results of using a stemless shoulder prosthesis in comparison with the results of using a conventional stemmed shoulder prosthesis in patients with primary osteoarthritis (OA) of the shoulder.

## Materials and methods

### Patients

The present prospective study included 82 patients with primary OA of the shoulder (stage III or IV according to Kellgren and Lawrence [22]) that was treated with primary shoulder arthroplasty between June 2006 and August 2009. All patients had symptoms for longer than 12 months before surgery and underwent a course of conservative treatment, including anti-inflammatory medication and home-based physical therapy. The indication for operative treatment was a persistent, severe, or moderate pain at rest and loss of shoulder function despite conservative treatment. The patients included in this study were divided into two groups: group 1 (standard anatomic shoulder prosthesis, Affinis^®^, Mathys, Bettlach, Switzerland) and group 2 (stemless shoulder prosthesis, TESS^®^, Biomet Inc., Warsaw, IN, USA). Both groups were matched according to age, gender, and follow-up. Patient assignment to each group was performed according to their medical record numbers. Patients with odd medical record numbers were placed in group 1 and patients with even medical record numbers were placed in group 2. Descriptive data for the patient groups are summarized in Table 1. No other significant neuromuscular or skeletal pathologies were present. None of the shoulders had undergone previous surgical procedures. None of these patients reported severe discomfort in the shoulder on the uninvolved side. The unaffected shoulder was examined clinically and showed no signs of severe impingement syndrome or OA. The study was performed in accordance with the ethical standards of the 1964 Declaration of Helsinki and was approved by the local ethical committee. Written informed consent was obtained from all patients.

**Table 1:** Demographic and clinical data for the patients, categorized by treatment group

	Group 1	Group 2	*P* value	*F* value
Number of patients	41	41	1.000	0.000
Gender (male/female)	14/27	14/27	1.000	0.000
Age (years) Range	67.05 ± 8.5 (45–79)	67.22 ± 9.0 (45–78)	0.390	0.747
Follow-up period (months) Range	32.7 ± 4.8 (24–47)	30.8 ± 4.6 (24–43)	0.691	0.159
Side (right/left)	22/19	23/18	0.671	0.181
Operative time (min) Range	106.2 ± 23.3 (60–165)	91.5 ± 14.5 (50–120)	0.002	10.054
Estimated blood loss (ml) Range	593.4 ± 147.0 (350–850)	496.3 ± 116.3 (200–800)	0.026	5.113
Hospital stay (days) Range	6.9 ± 0.82 (5–9)	6.7 ± 0.72 (5–8)	0.633	0.229

*Group 1* stemmed shoulder prosthesis, *group 2* stemless shoulder prosthesis

Data are given as the mean ± standard deviation

### Surgical technique

All operations were performed by the authors with the patients in a beach chair position under general anesthesia in combination with an interscalene block via a standard deltopectoral approach. All humeral stems in group 1 were inserted with cement. In all patients, glenoid resurfacing was performed with a cemented all-polyethylene glenoid (group 1: two-pegged glenoid component; group 2: a keeled glenoid component). Due to the presence of degenerative changes in the biceps tendon (inflammation, partial rupture, instability) in both study groups, a biceps tenotomy or tenodesis was always performed (group 1: 30 tenotomies, 11 tenodeses; group 2: 27 tenotomies, 14 tenodeses).

### Postoperative rehabilitation

After surgery, all patients were immobilized in a sling, and they gradually discontinued using it over a period of five weeks. All of patients underwent the department’s standard inpatient rehabilitation program during the hospital stay, and an additional outpatient rehabilitation program continued for six weeks after discharge. This outpatient rehabilitation program was standardized and similar for both groups. Passive mobilization and active assisted exercises within the pain-free range of motion were also performed up to six weeks after surgery. Afterwards, active exercises with and without resistance were initiated. All patients were treated with continuous passive motion within the first three weeks. Additionally, for all patients, physical therapy was provided in our institution’s outpatient rehabilitation unit for about six months to strengthen the shoulder and maximize the range of motion until there was maximum improvement.

### Evaluation

All patients were evaluated preoperatively using radiographs of the shoulder (true anteroposterior view with the humerus in neutral rotation, and axial and scapular views to confirm the presence of bony changes). If necessary, an additional MRI of the shoulder was performed to assess the status of the rotator cuff. Immediately after surgery, the patients had true anteroposterior radiographs taken with neutral rotation of the humerus. True anteroposterior radiographs with neutral rotation of the humerus and axillary radiographs were obtained during the follow-up to assess component position and to check for signs of prosthetic loosening.

The operative record, the anesthesia record, and the nursing notes were analyzed to estimate blood loss during and after the surgery.

The subjects were assessed clinically before and after surgery using the Constant score [23] and the DASH score [24]. In addition, the range of motion (abduction, anteversion, external rotation in neutral arm position) was assessed with a goniometer. Complications were noted. At follow-up, symptoms were assessed by performing an interview, and all patients were clinically examined by the authors.

### Statistical analysis

We used an analysis of variance for repeated measures to detect possible differences between the two treatment groups during the follow-up, and a post hoc LSD test where appropriate. The intra-subject factor was time (preoperative, postoperative) and the inter-subject factor was the study group (conventional anatomic shoulder prosthesis, stemless shoulder prosthesis). We used the parametric unpaired *t* test to compare the preoperative values within the treatment groups. A significance level of <0.05 were assumed. We used SPSS statistical software (version 18.0 for Windows) for all calculations. Unless specified otherwise, results are given as the mean ± standard deviation.

## Results

### Subjects

There were no significant differences between the groups with regard to age, gender, and follow-up (Table 1). The preoperative functional status (Constant score, Dash score and range of motion) of the patients are presented in Table 2. Except for abduction (*P* < 0.001, *F* = 22.058), anteversion (*P* = 0.003, *F* = 9.386), and consequently the category “motion” of the Constant score (*P* < 0.001, *F* = 23.158), the preoperative values for active range of motion, Constant score, and Dash score did not differ significantly between the treatment groups.

**Table 2:** Preoperative and postoperative values of Constant score, DASH score, and range of movement for the entire series

	Preoperative	Postoperative	*P* value	*F* value
Pain (points)				
Group 1	1.7 ± 2.4	9.5 ± 3.0	0.904	0.015
Group 2	1.8 ± 2.4	9.7 ± 2.5		
All	1.7 ± 2.4	9.6 ± 2,7		
Activity (points)				
Group 1	5.7 ± 2.2	11.0 ± 3.5	0.465	0.539
Group 2	6.4 ± 1.9	12.2 ± 2.0		
All	6.0 ± 2.1	11.6 ± 2.9		
Motion (points)				
Group 1	15.5 ± 3.1	22.0 ± 4.3	0.551	0.359
Group 2	17.9 ± 5.3	25.0 ± 5.4		
All	16.7 ± 4.5	23.5 ± 5.1		
Strength (points)				
Group 1	3.3 ± 2.3	6.3 ± 3.7	0.197	1.695
Group 2	3.9 ± 2.1	7.7 ± 2.7		
All	3.6 ± 2.2	7.0 ± 3.3		
Total (points)				
Group 1	26.3 ± 5.7	48.9 ± 7.4	0.185	1.784
Group 2	30.1 ± 7.1	54.7 ± 7.3		
All	28.2 ± 6.7	51.8 ± 7.9		
Total adjusted (points)				
Group 1	34.9 ± 8.0	65.0 ± 11.0	0.162	1.99
Group 2	40.1 ± 9.5	73.2 ± 11.3		
All	37.5 ± 9.1	69.1 ± 11.8		
Dash (points)				
Group 1	63.2 ± 12.5	47.3 ± 12.4	0.592	0.289
Group 2	62.1 ± 11.6	47.4 ± 12.1		
All	63.2 ± 12.5	47.4 ± 12.4		
Anteversion (°)				
Group 1	72.8 ± 12.1	103.3 ± 14.1	0.076	3.234
Group 2	81.2 ± 6.6	115.9 ± 9.8		
All	77.1 ± 10.6	109.6 ± 13.7		
Abduction (°)				
Group 1	63.0 ± 13.7	96.9 ± 14.0	0.283	1.168
Group 2	68.2 ± 6.7	105.0 ± 12.1		
All	65.6 ± 11.0	101.0 ± 13.6		
External rotation (°)				
Group 1	30.1 ± 9.3	48.6 ± 11.0	0.141	2.214
Group 2	39.1 ± 11.1	54.4 ± 10.7		
All	34.6 ± 11.3	51.5 ± 11.2		

*Group 1* stemmed shoulder prosthesis, *group 2* stemless shoulder prosthesis

Data are given as the mean ± standard deviation

### Clinical assessment

There was a main effect of time on all directions of the active range of motion (*P* < 0.001), on all categories of the Constant score (*P* < 0.001) and on the Dash score (*P* < 0.001, *F* = 207.89), which suggests that the postoperative results obtained following both types of shoulder arthroplasty yielded highly significant improvements over the preoperative values. We could not detect a significant interaction between the patient groups and time during the follow-up with respect to the Constant score, Dash score, and active range of motion (Table 2). This indicates that the increases in range of motion and functional status (Constant score, DASH score) after surgery in patients treated with either standard anatomic shoulder prostheses or stemless shoulder prostheses were comparable. Likewise, the mean hospital stay after surgery did not differ significantly between the treatment groups (Table 1). In contrast, the mean operative time was significantly higher in patients treated with cemented stemmed shoulder prostheses than in patients treated with stemless shoulder prostheses without cemented fixation of the humeral implant (Table 1).

In addition, the estimated blood loss was also significantly higher in group 1 than in group 2 (Table 1). However, no patient in either study group required a peri- or postoperative blood transfusion.

### Radiological assessment

There were no radiolucent lines around the humeral implants or osteolyses. In four patients in the stemmed shoulder arthroplasty group and three patients in the stemless shoulder arthroplasty group, we were able to detect mild glenohumeral subluxation, which means that there was anterior translation of <25 % with respect to the amount of translation of the center of the prosthetic head relative to the center of the glenoid.

We could detect implied radiolucent lines around the glenoid component in seven patients in the stemmed shoulder arthroplasty group and nine patients in the stemless shoulder arthroplasty group without any signs of migration or loosening of the glenoid. Despite the radiological findings and moderate pain, none of the patients required reoperation during the follow-up.

### Complications

Intraoperative complications included one fissure of the glenoid in group 2 and a fissure of the greater tuberosity in group 1 which healed without the need for additional treatment. Postoperatively, a hematoma of the shoulder developed in one patient in group 1, which resolved without operative treatment (an aspiration was performed). Furthermore, one superficial wound infection was treated successful using oral antibiotics in group 1. One patient in group 2 had a temporary incomplete brachial plexus neuropathy, possibly caused by a local hematoma after the plexus anesthesia, which resolved two months after surgery.

## Discussion

The results afforded by new shoulder implant systems must be compared with the good results obtained using the existing concepts for shoulder arthroplasty. The use of modern anatomically designed shoulder implant systems in combination with glenoid resurfacing provides significant pain relief and improvement of function over the long term [25]. However, the clinical outcome and the long-term survival of the prosthesis in existing modern shoulder prosthesis systems can be affected in particular by problems with the soft tissue and complications with the glenoid component [16, 26, 27]. In contrast, complications relating to the humeral component (such as loosening and malpositioning of the humeral stem or periprosthetic fractures) are much less common (an incidence of approximately 1 % according to the literature); however, given the rising numbers of shoulder implants that are being performed, they are clinically relevant [16]. Another problem with the use of a standard stemmed shoulder prosthesis can occur in patients with posttraumatic situations of the proximal humerus (fracture sequelae). These cases are often associated with deformities of the proximal humerus, especially in the area of the humeral metaphysis. Therefore, the correct positioning of the humeral stem can be challenging and technically demanding [28, 29], which is clearly reflected in the complication rate of 25–32 % [30–32]. A frequent complication after total shoulder arthroplasty is postoperative tearing of the rotator cuff, which may also result in revision of the anatomical total shoulder replacement and the implantation of a reversed shoulder arthroplasty [16]. In these cases, the removal of the humeral stem is also associated with potential problems like fractures with the need for a new massive humeral stem [16]. An alternative that avoids these typical humeral problems is the use of humeral head resurfacing. Nevertheless, surface replacement arthroplasty is also potentially associated with typical problems, such as the potential risk of “overstuffing” the humeral head component; the use of a glenoid component is also technically demanding as the humeral head is left in situ [33].

Due to these specific problems, the anatomic stemless shoulder prosthesis was developed in the mid-1990s. The characteristic feature of this type of prosthesis is the cementless metaphyseal fixation of the implant by some mechanism. The essential advantage of this concept is the fixation of the humeral component without the need to prepare the humeral diaphysis. Therefore, the humeral head can be positioned regardless of the shape of the humeral diaphysis. Aside from patients with primary OA, this fixation technique is particularly useful in patients with posttraumatic OA of the shoulder and deformities in the metaphyseal region.

Another advantage of the stemless shoulder prosthesis is the preservation of the humeral bone stock. This potentially results in better starting conditions if any revision surgery is needed, and reduces the potential risk of typical complications such as intraoperative humeral fracture in cases requiring removal of the humeral stem. Furthermore, in comparison with humeral head resurfacing, adequate exposure of the glenoid to allow the implantation of a glenoid component is much easier to achieve.

In this study, we compared the clinical and radiological results of patients with primary OA of the shoulder who were surgically treated with either a standard stemmed shoulder prosthesis or an anatomical stemless shoulder prosthesis. To our knowledge, this is the first study to be age, gender, and follow-up matched in order to allow the results obtained with both types of prosthesis in patients with primary OA of the shoulder to be compared. In accordance with several other studies [25, 34], our results show significant improvement in shoulder function and pain relief across the entire investigation series. In particular, the clinical results noted here for the stemless shoulder prosthesis were comparable with those reported by other authors [19–21]. However, we did not detect a significant difference in the range of movement, DASH score, and Constant score between both types of prosthesis within the follow-up period. Otherwise, with respect to the intra- and perioperative data, their shorter operative times and reduced loss of blood represent potential advantages of the patient group treated with stemless shoulder prosthesis.

However, another possible reason for the shorter operative time and the minor blood loss seen when using the stemless shoulder prosthesis could be that this is due to the use of different implant fixation techniques in both treatment groups. In contrast to the cementless fixation of the stemless shoulder prosthesis, all conventional humeral stems in the other treatment group were inserted with cement, which necessitated additional operative time for the cement to harden. In addition, patients receiving a standard anatomic shoulder prosthesis (group 1) required more extensive preparation of the humerus, which can lead to higher blood loss. Despite this potential benefit of the stemless shoulder prosthesis, no clinical consequences of it, such as blood transfusion or a higher revision rate in group 1 due to perioperative complications, were observed: the results of radiological investigations and the complication rates in both groups were comparable.

Therefore, we can conclude that the stemless shoulder prosthesis provides good clinical results in patients with patients with OA of the shoulder without the need for a humeral stem. This surgical technique offers a reliable method for the anatomic restoration of the proximal humerus. The metaphyseal anchorage of the humeral implant used in this study was achieved using a specially designed fixation device with six fins coated with hydroxyapatite of different sizes. The impaction of this fixation system with automatic centering in the metaphyseal region of the humerus is appropriate in the normal bone structure as well as in the soft bone structure. Beside the configuration of the spongiosa, the humeral head must be cut precisely such that a plane and stable surface of the bone is obtained for sufficient osteointegration of the implant. If required, an additional bone graft from the resected humeral head can be placed below the prosthetic head, improving the primary stability of the implant. If primary stability of the humeral implant cannot be realized, the placement of a stemmed shoulder prosthesis is mandatory during the surgery as an alternative option. If necessary, a preoperative CT investigation may be helpful for assessing the bone quality of the proximal humerus with respect to the implantation of a stemless shoulder prosthesis.

Although investigating the influence of the factor age on the clinical results was not a main aim of our study, the results presented here indicate that the implantation of a stemless shoulder prosthesis represents a reliable option for surgical treatment, even in elderly patients with potentially decreased bone osseous mineral density. Another influence on the functional results after total shoulder arthroplasty is the status of the rotator cuff [3], which also often shows considerable lesions, particularly in elderly patients. Therefore, when necessary, an additionally MRI investigation was carried out in these patients to exclude massive rotator cuff tears. We were also able to demonstrate [35] that the implantation of a stemless shoulder prosthesis as a hemiarthroplasty can also yield reasonable results in a patient with a rotator cuff tear arthropathy and low functional demands.

One limitation of our study was the duration of our follow-up. Our assessment at 32 months prevents any conclusion from being drawn regarding the long-term results, especially those for the new anatomical stemless shoulder prosthesis. We cannot exclude the possibility that our follow-up was too short to detect possible differences in the survivorship of this prosthesis.

In conclusion, this study demonstrates good midterm results following the placement of an anatomically designed stemless shoulder implant system in the treatment of OA of the shoulder. In addition, the technical aspect of the metaphysal fixation allows precise and simple reconstruction of the proximal humerus with preservation of the humeral bone stock and adequate exposure of the glenoid. Further investigations are needed to determine the long-term performance of this prosthesis.
